# Supplementary material for: A Simple Method to Establish Sufficiency and Stability in Meta-Analyses: With Application to Fine Particulate Matter Air Pollution and Preterm Birth
Source: Int J Environ Res Public Health. 2022 Feb 11;19(4):2036. doi: 10.3390/ijerph19042036 (PMC8871712; doi:10.3390/ijerph19042036)
Supplement: Supplementary file 1 [file ijerph-19-02036-s001.zip › ijerph-1504259-supplementary.pdf]

Supplementary Materials

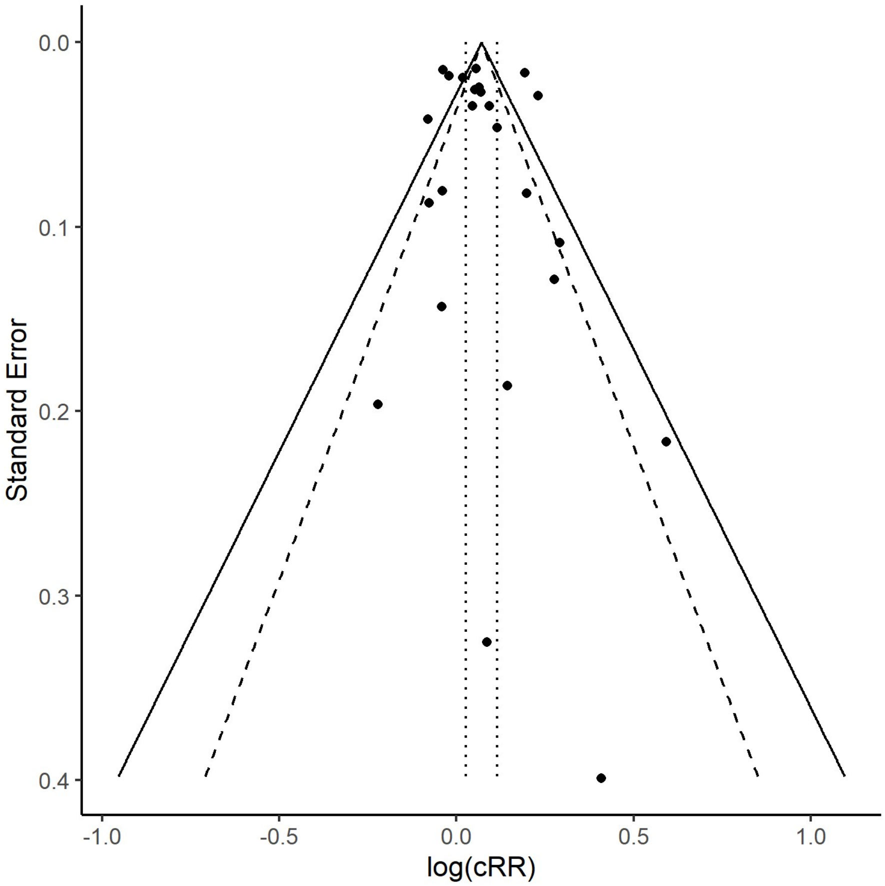

Figure S1: Funnel plot with 99% confidence interval limits (continuous lines) and 95% confidence interval limits (dashed lines) for the funnel, with 95% confidence interval bars for the logarithm of the cumulative relative risk (cRR).

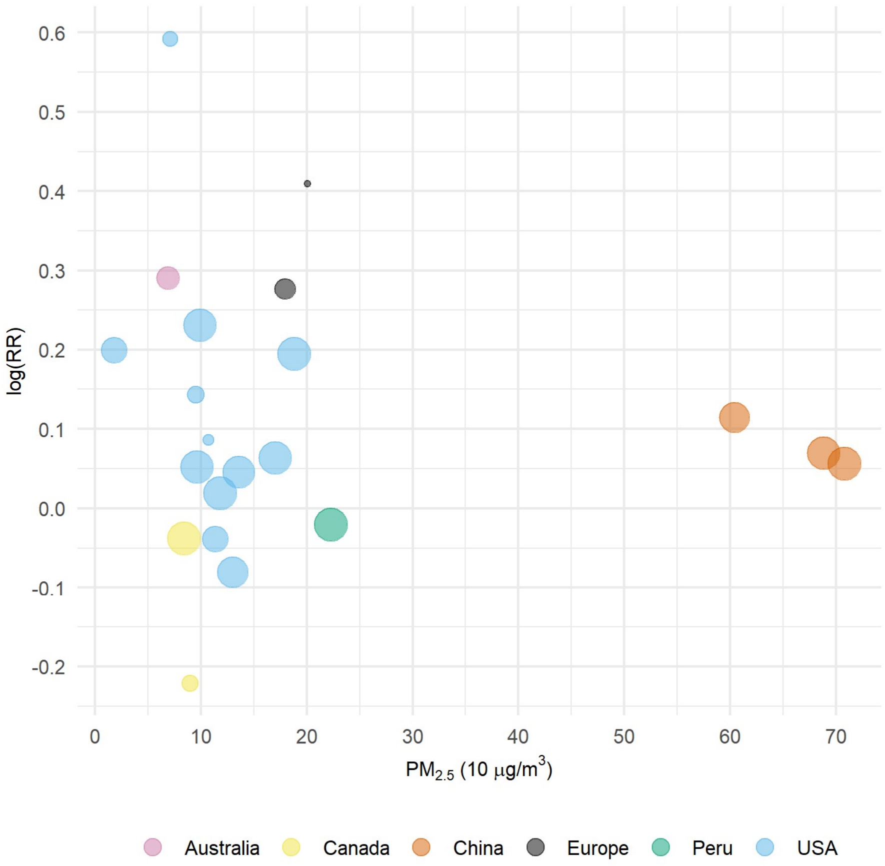

Figure S2: Log relative risk (cRR) of preterm birth (PTB) by whole-pregnancy mean exposure to fine particulate matter ($PM_{2.5}$) by geographic region. Sizes of the points are proportional to the weights included in the random effects meta-analysis.
